# Endoscopic Removal of a Supernumerary Premolar in the Mandible during a Dental Implant Placement

**DOI:** 10.1155/2014/975470

**Published:** 2014-03-19

**Authors:** Víctor Beltrán, Mario Cantín, Eduardo Borie, Ramón Fuentes, Wilfried Engelke

**Affiliations:** ^1^CIMOFIR Research Center, Dental School, Universidad de La Frontera, Manuel Montt 112, 4781176 Temuco, Chile; ^2^Dentistry Centre, Department of Maxillofacial Surgery, Georg-August-Universität, Robert Koch Street 40, 37099 Göttingen, Germany; ^3^CIMA Research Center, Dental School, Universidad de La Frontera, Manuel Mont 112, 4781176 Temuco, Chile; ^4^Dental Materials and Prosthodontics Department, Ribeirão Preto Dental School, University of São Paulo, Avenida Café w/n, 14040-904 Ribeirão Preto, SP, Brazil

## Abstract

The surgical removal of supernumerary teeth is necessary in some cases, especially before the commencement of any orthodontic or implant treatment procedure. In the mandibular supernumerary premolar, a more conservative approach is required because of the presence of complications associated with conventional surgery due to the close proximity of the said premolar to the alveolar inferior and mental nerves, and the need for bone conservation for implant placement. The endoscopic surgical approach has been used for the removal of the maxillary supernumerary tooth, impacted third molar, and implants. In this case report, we present an endoscopically assisted surgical technique for the removal of an unerupted supernumerary premolar in the mandible associated with a dental implant placement procedure.

## 1. Introduction

A supernumerary tooth is defined as any tooth or odontogenic structure formed from a tooth germ in excess of the usual number in any region of the dental arc [1]. Supernumerary teeth have been found in all areas within the dental arches, as well as outside them, in primary and permanent dentition. They may be of single, multiple, unilateral, or bilateral distribution [2], with a prevalence in permanent teeth of between 0.15% and 3.9% [3]. It is classified morphologically into conical, tubercles, complementary, and odontoma types. Mesiodens is the most common type in terms of location, followed by paramolars in the premolar area [3].

Supernumerary teeth are the result of changes occurring in the process of normal epithelial-mesenchymal interactions of tooth development. However, the etiology of supernumerary teeth is unknown. Some of the most accepted theories suggest the dichotomy of the tooth germ, other overgrowth, or hyperactivity of the dental lamina, where the proliferations of epithelial rests of dental lamina induced by pressure from the rest of the dentition are outbreaks of supplemental supernumerary teeth [2].

The diagnosis is easy when supernumerary teeth are erupted. However, many do not erupt and may remain asymptomatic throughout life. Some cases are responsible for disorders such as delayed tooth eruption, tooth malposition, or associated pathologies such as dentigerous cyst, requiring surgical intervention [2]. The finding of an unerupted supernumerary tooth in a potential site for the placement of implants can make a forecast difficult; prior removal of the supernumerary tooth with removal of the surrounding bone for broad access will be required, generating major defects after removal.

The use of support endoscope makes a minimally invasive and more predictable procedure possible, in terms of greater conservation of bone tissue, less tissue damage, and minimization of blood loss [4, 5]. Some authors have reported and recommended its use for the removal of ectopic teeth located on sites such as the nasolacrimal duct, maxillary sinus, nasal fossa, and condyle; for the removal of implants displaced into the maxillary sinus [6, 7] and for the removal of ectopic third molar [8] and lesions such as ameloblastic fibroodontoma [9] or schwannoma. In this case report, we present an endoscopically assisted surgical approach for the removal of an unerupted supernumerary premolar during endosseous implant placement in the posterior left mandible.

## 2. Case Description

**Figure 1:**
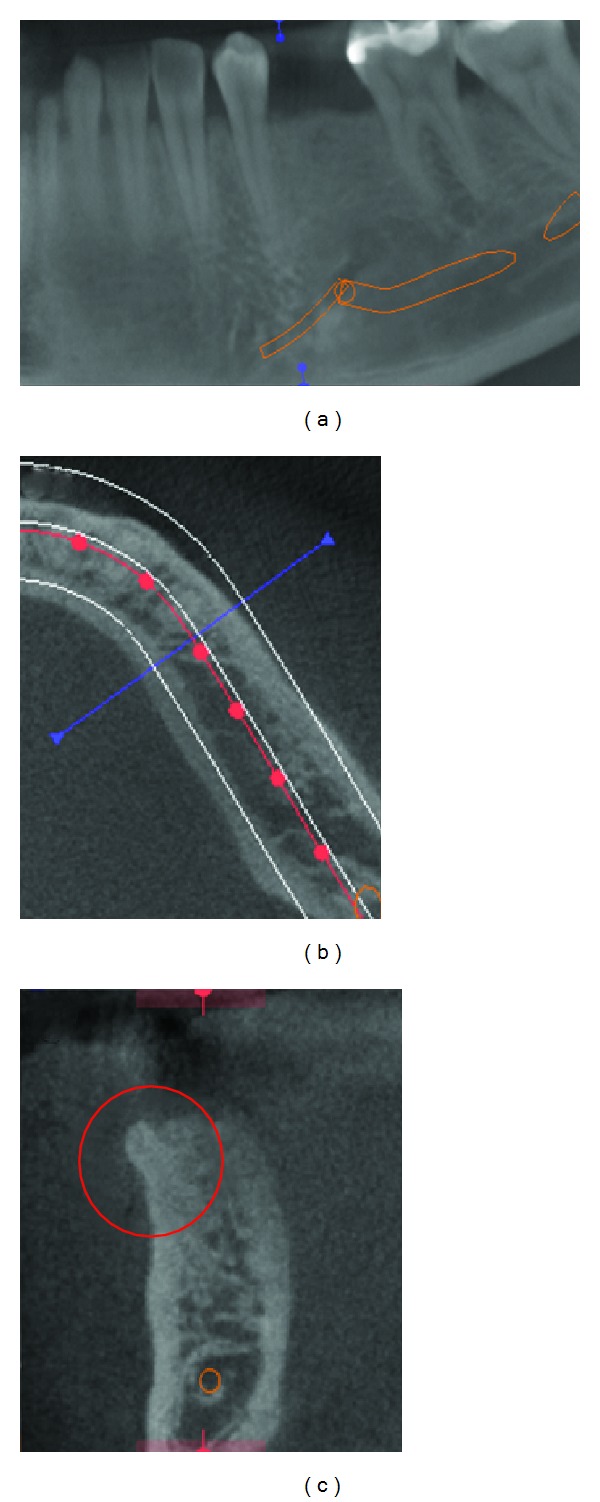
Panoramic radiograph. Note that it is aware of the presence of supernumerary tooth (a). Location through computed tomography. The blue line illustrates the parasagittal section which locates the supernumerary tooth (b). Supernumerary tooth (red circle) located in relation to the buccal cortex (c).

**Figure 2:**
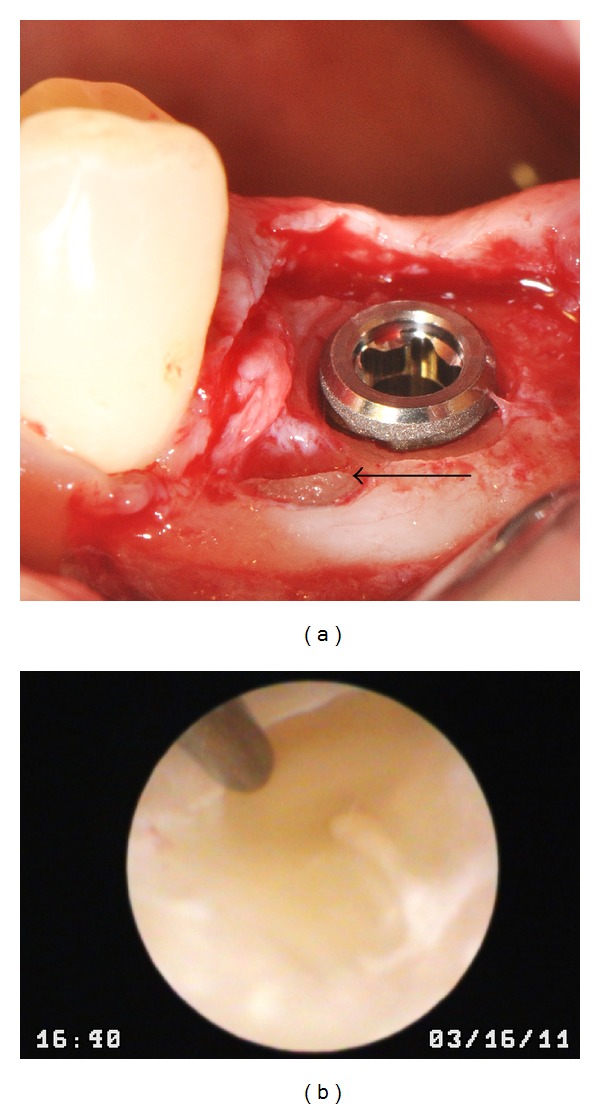
Removal by milling the coronal portion of the supernumerary tooth ankylosed to the vestibular cortex. Note the mesial and buccal relationship to the implant placed according to plan surgery. Black arrow shows the visualization of the supernumerary tooth crown near the buccal cortex after superficial osteotomy (a). Immersion endoscope (IE) visualization during the drilling of the radicular portion of the supernumerary tooth, observed with a yellowish color and apparently had two fused roots in an anterior portion (b).

**Figure 3:**
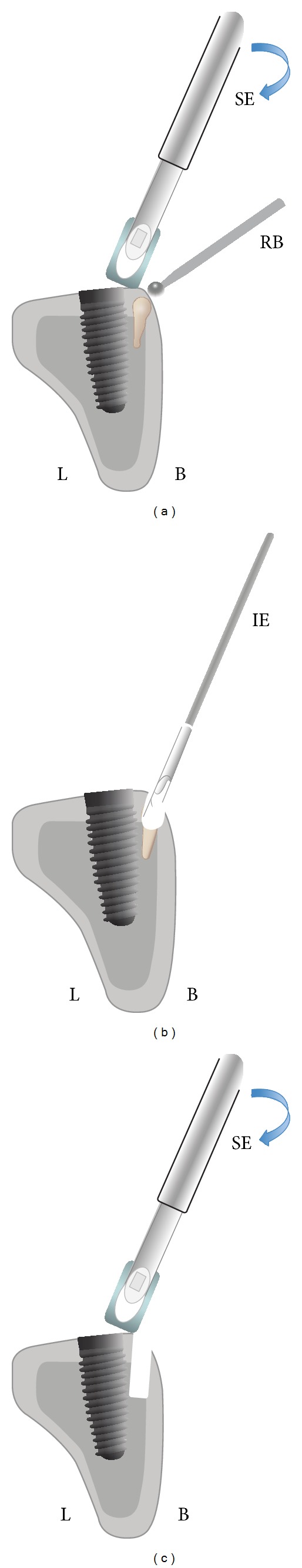
Schematic diagram of supernumerary tooth removal with endoscopic approach. (a) Support endoscopy (SE), an endoscope of 2.7 mm diameter and a 30-degree view angle, with support and irrigation, was used for drilling of the tooth. (b) Immersion endoscopy (IE), an endoscope of 1.9 mm diameter, with 30- and 70-degree view angles and continuous irrigation via the support sheath, was used, which permits either short distance observation or up to 40x visualization when there is direct contact with the site by saline solution. (c) Complete elimination of the tooth structure without affecting the bone wall. The bone defect created was inspected with SE. No communication with the implant and buccal bony wall fully retained was observed.

**Figure 4:**
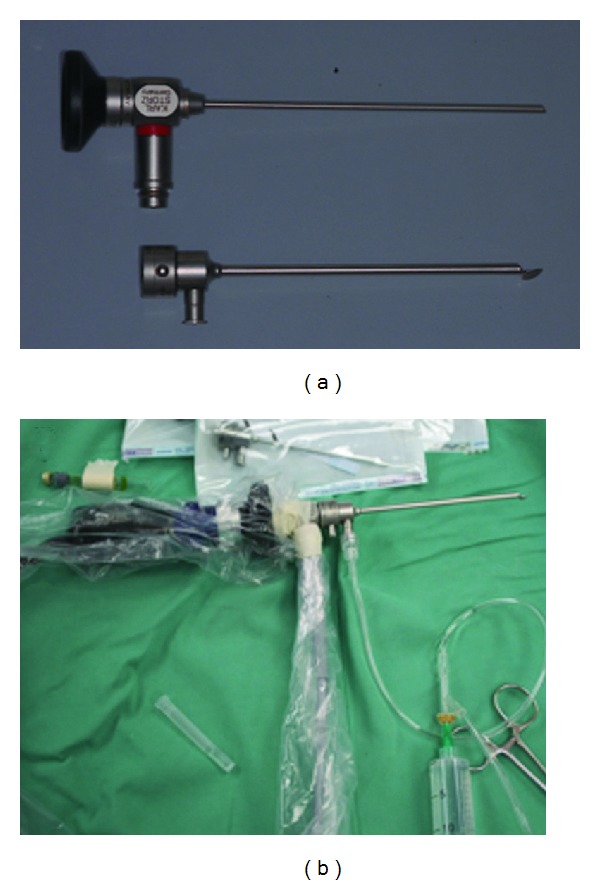
Endoscopic visualization techniques in oral surgery and implantology. (a) Support Endoscopy (SE). A Karl Storz optic (2.7 mm diameter) with support and irrigation sheath. (b) Immersion endoscopy (IE). Sterile equipment, Karl Storz optic (1.9 mm diameter) with supporting sheath to accommodate the 1.9 mm diameter optic connected to saline irrigation, tip modified to adapt to the dental alveolus.

A 34-years-old man, systemically healthy, was underwent surgery at the Oral Microsurgery Center, Dental School, Universidad de La Frontera. The patient has given their informed consent for the case to be published. We planned the placement of one titanium osseointegrated-type implant in the edentulous left posterior mandible, in the premolar (3.5) area. In the review of the preoperative panoramic radiograph, no supernumerary tooth in the surgical area was observed (Figure 1(a)). Nevertheless, we found a diffuse supernumerary tooth near the buccal cortex in the parasagittal serial section analysis of the area for implant placement (Figures 1(b) and 1(c)), with a similar appearance, small and obliquely to the buccal cortex, at a lower premolar. It was decided that a full-thickness mucoperiosteal flap would be performed, noting the bone to implant surface, with direct visualization of the edge of the supernumerary tooth crown near the buccal cortex. The implant (3.7 mm diameter and 11 mm length) was placed in the second premolar area previous to the supernumerary tooth removal to ensure the optimal primary stability (Figure 2(a)). To access the supernumerary tooth, an endoscopic approach was performed (schematic representation in Figure 3). The endoscopic equipment consisted of rigid endoscopes of 2.7 and 1.9 mm diameter with support and irrigation sheaths (Karl Storz, Tuttlingen, Germany) (Figure 4). The endoscopes were linked up with a Storz 487 B examination unit and a Xenon 300 W light fountain with 6000 K capacity (Karl Storz, Tuttlingen, Germany).

**Figure 5:**
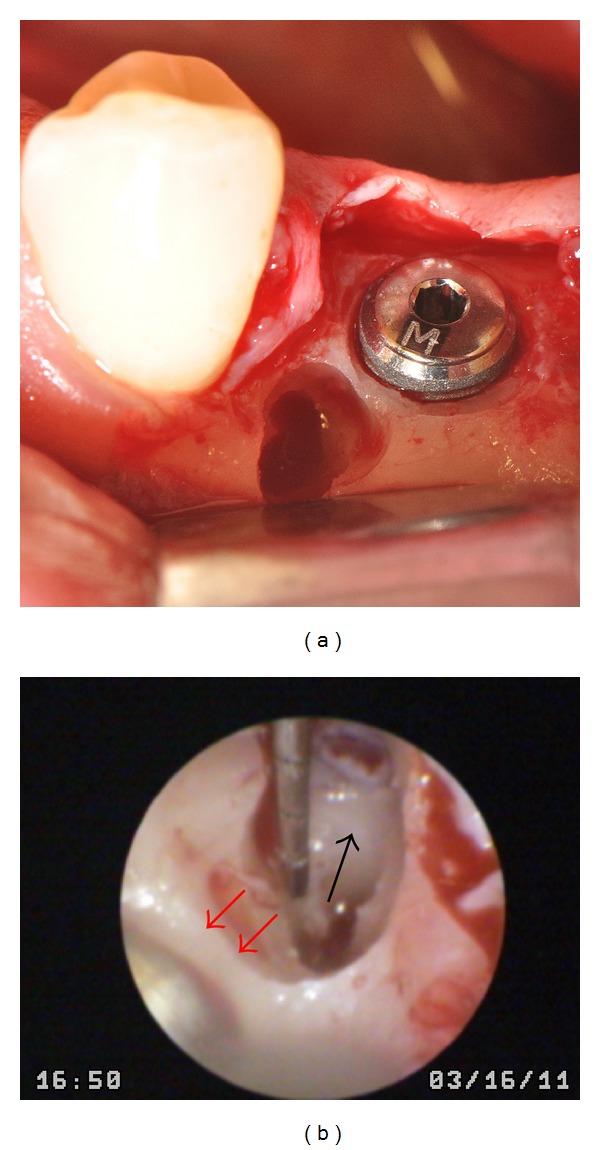
Total removal of supernumerary tooth (a). Support endoscope (SE) or macroscopic endoscopic control; note the integrity of the buccal cortex (red arrows) and no communication with the implant (black arrow) (b).

For support endoscopy (SE), an endoscope of 2.7 mm diameter and a 30-degree view angle, with support and irrigation, was used for drilling of the tooth. Later for immersion endoscopy (IE) [10], an endoscope of 1.9 mm diameter, with 30- and 70-degree view angles and continuous irrigation via the support sheath, was used, which permits either short distance observation (2-3 mm) or microscopic (up to 40x) visualization when there is direct contact with the site facilitated by the endoscope's terminus being immersed in saline solution (Figure 2(b)) [11, 12]. We eliminated completely the tooth structure without affecting the bone wall. The bone defect created after the supernumerary tooth removal was inspected with SE. No communication with the implant was found nearby, with the buccal bony wall fully retained and no remaining tooth structure (Figure 5). The procedure for access to the supernumerary tooth and its removal under SE and IE took approximately 25 minutes with minimal bleeding.

**Figure 6:**
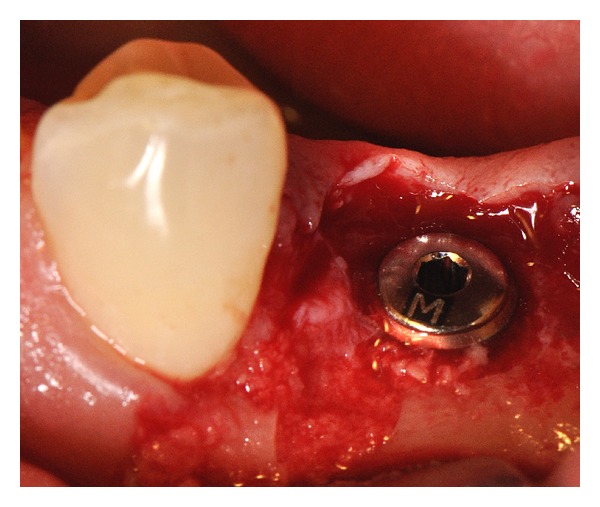
Four-wall bony defect filled with particulate bone graft.

Finally, the bone defect was filled with particulate bone graft (Figure 6), with the flap repositioned and sutured. Postoperatively, meloxicam 15 mg/day was only indicated for three days. The postoperative patient was excellent and uneventful. Sutures were removed seven days after surgery, and healing was satisfactory.

## 3. Discussion

The use of endoscopy in oral and maxillofacial surgery has improved the visualization of the surgical field through the magnification of the operative field, with lighting and additional irrigation attached to the support, making possible minimally invasive and conservative approaches with precise dissection. These characteristics make it an ideal alternative for the elimination of unerupted supernumerary teeth [4, 9].

The occurrence of supernumerary teeth is not unusual. It is associated with more than 20 syndromes and different growing conditions and nonsyndromic conditions. Premolars are often asymptomatic, and most cases are diagnosed as imaging test findings before orthodontic or implant placement treatments. Only 2% of premolars are likely to generate pathological conditions as a dentigerous cyst or the adjacent tooth root resorption. In many cases, surgical removal of these teeth is the only treatment option, with the complex vision of the surgical site requiring the surgeon to remove a large amount of bone tissue, which can cause neurosensorineural complications when the teeth are very close to the inferior alveolar and mental nerves. Moreover, due to the complex morphology of these teeth, there are potential risks of leaving remnants of dental tissues and dental sac. In addition, supernumerary teeth are not always implemented in the direction of normal eruption; they can appear upside down or cross an ectopic or abnormal eruption path, making their removal even more complex.

It has been reported that the presence of unerupted supernumerary in a potential implant site may compromise the placement of implants and bone blocks, thus requiring their prior removal. If the unerupted supernumerary is removed at the same time of implant placement, the use of bone grafts may be necessary in the defect created [2], or it might be necessary to delay implant placement in another surgical procedure. In a series of five case reports Davarpanah and Szmukler-Moncler [13] suggest that implants placed in contact with ankylosed root fragments might not interfere with implant integration, at least in the mid-term, where appearance of the bone-implant interface was similar to osseointegrated implants on periapical radiographs. But a durable osseointegration can be gained only through a direct bone–implant contact, without interposition of fibrous tissue or any other root material [14]. In this way Guarnieri et al. [15] provided the histology of a human root-implant interface with the formation of anormal tissues around the implant described as osteocementum or dystrophic cementum.

Several cases have demonstrated the advantages of endoscopic surgery. Hasbini et al. [6] reported the case of an ectopic third molar in the maxillary sinus, which endoscopic surgery addressed at the osteomeatal complex (Caldwell-Luc). Suarez-Cunqueiro et al. [8] reported the first case of an endoscopic surgical technique by support endoscope for the removal of an ectopic third molar in the condylar process of the mandible associated with a dentigerous cyst. Nakamura et al. [5] reported the endoscopic removal of a dental implant displaced into the maxillary sinus, with the endoscopic surgical approach described as being reliable and minimally invasive. Sanei-Moghaddam et al. [7] presented the case of a supernumerary erupted into the nasal cavity that was removed endoscopically. Franco et al. [9] reported an endoscopic removal of an ameloblastic fibroodontoma in the mandible of a child by enucleation with a nasal endoscope, which allowed them to ensure that the entire lesion had been removed; there was also minimal bone resection, which reduces the risk of growth disorders.

Another benefit observed in our case, aside from the optimal viewing at any time of the surgery with SE, was the minimal and controlled removal of bone tissue, with the immediate postoperative control of clean bedding without remaining tooth structure (with IE). Therefore, we obtained a four-wall defect without communication to the implant and preservation of the buccal cortex, thus improving the prognosis of the implant, especially when a tooth ankylosed, it is very difficult to define its margin, where SE and IE show superiority ensuring complete dental tissue removal.

Nevertheless, some considerations are necessary for this procedure. First, the technique requires a core team of endoscopic and specially instructed surgeons. The endoscope provides a magnified image of two dimensions on a video monitor at a distance, thus requiring the development of specific hand-eye coordination, with a broad understanding of the three-dimensional concept of oral and maxillofacial surgical anatomy [8]. Second, it has limited its use to when the purpose of removal is large [5]; a situation that is overcome by the combination of SE macroscopic optimized for controlled milling of the tooth in relation to bone, with IE that allows microscopic visualization of 40x magnification for detailed discrimination of hard and soft tissue, minimizing the level of risk of the procedure [16].

## 4. Conclusion

We suggest the use of an endoscopic surgical approach as a first-line treatment for the removal of supernumerary teeth adjacent to the implant area, to preserve bone and prevent possible damage to neurovascular structures or implant viability. This endoscopic removal technique can be applied for unerupted supernumerary mesiodens in the maxilla that is the most common type.
